# Effect of sex ratio on the life history traits of an important invasive species, *Spodoptera frugiperda*

**DOI:** 10.1515/biol-2022-0873

**Published:** 2025-02-25

**Authors:** Su-Ran Wu, Hui Wang, Chun-Jie Zhao, Yan Xiong, Jun-Hua Ren

**Affiliations:** Institute of Tropical Bioscience and Biotechnology, Chinese Academy of Tropical Agricultural Sciences, Haikou, 571101, PR China; College of Bioengineering, Henan University of Technology, Zhengzhou, 450046, PR China

**Keywords:** *Spodoptera frugiperda*, sex ratio, longevity, fecundity, reproduction

## Abstract

The fall armyworm (FAW), *Spodoptera frugiperda*, is a dangerous migratory pest. Evaluating the effect of sex ratio on the FAW offspring population is particularly important for field control. In this study, five different sex ratio treatments (female/male = 3:1, 2:1, 1:1, 1:2, and 1:3) were conducted to investigate the effects of sex ratio on the life history traits of FAW. The results showed that sex ratio significantly affected lifetime fecundity, developmental duration of the preadult stage, hatch rate, and emergence rate but had no effect on longevity of parental and offspring adults, larval duration, pupation rate, or number of eggs/moth of offspring. The lifetime fecundity and hatch rate of parental adults and the number of adult offspring/moth were the lowest when the sex ratio was 3:1, while the lifetime fecundity and number of adult offspring/moth were the highest and pupation duration was the shortest when the sex ratio was 1:1. The number of eggs/moth of parental adults and total adults in the F1 generation were higher in male-biased groups than in female-biased groups, and male annihilation appears to be a more effective control strategy. These findings have implications for improving laboratory rearing, population forecasting, and control of FAW in the field.

## 1 Introduction

*Spodoptera frugiperda* J. E. Smith, 1797 fall armyworm (FAW) is a globally recognized pest native to the tropical and subtropical regions of the Americas [1]. This species has become a significant threat to agriculture worldwide due to its broad host range, high fecundity, robust migratory ability, and high resistance to insecticides [2]. Since its initial invasion in Yunnan, China, in late 2018, FAW has spread to 26 Chinese provinces, causing significant damage to both cereal crops and a variety of economic crops [3,4]. According to their feeding preference on their hosts, the armyworm is divided into two biological types: the “corn type” and the “rice type” [5]; the corn type FAW prefers corn, sorghum, sugarcane, etc., while the rice type prefers rice. The grassland armyworms that occur in China are all of the “corn type” [6].

The sex ratio plays a pivotal role in the management and control of FAW by influencing the insect’s reproductive potential, life cycle, and population dynamics of the insect; for example, the fecundity of *Assara inouei* Yamanaka (Lepidoptera: Pyralidae) first increases and then decreases as the proportion of male or female adults increases [7]. The oviposition period and fecundity of *Spodoptera exigua* (Hübner) (Lepidoptera: Noctuidae) increase with increasing sex ratio, while the egg hatch rate decreases significantly [8]. A high or low sex ratio hinders the reproduction of *Loxostege sticticalis* because the former shortens the lifespan of male and female moths, and the latter hinders the mating of males [9]. Sex ratio can be an important factor and shows variable relationships with the life history traits of different insects, which is the basis for pest prediction and control. Controlling the growth, fecundity, and population structure of FAWs can significantly reduce their threat to crops. However, the effect of sex ratio on the fecundity and population structure of adults and offspring of *S. frugiperda* has not been reported.

This study focuses on the effects of sex ratio on the fertility and population structure of adult FAW and their offspring. By conducting experiments with different sex ratio treatments, we compared developmental parameters such as adult longevity, fertility, pupation rate, and emergence rate. These findings are expected to contribute to the refinement of laboratory-rearing practices and improve the effectiveness of FAW population forecasting and field control.

## 2 Materials and methods

### 2.1 Insect rearing

The sex recognition of *S. frugiperda* pupae and adults was followed as Dong’s method [10]. For adults, there is a large white spot on the tip of the male wing, a light-colored band on each side of the circular pattern, and a white wedge-shaped pattern on the inner side of the kidney-shaped spot. The abdomen of the adult male was long and narrow with long yellow tail hairs at the end, while the abdomen of the adult female was coarse and cylindrical with a cluster of short yellow tail hairs.

In June 2021, third instar larvae of *S. frugiperda* were obtained from a sugarcane field located in Chengmai, Hainan Province, China (Long, Lat:110.086595,19.84839). These larvae were reared on a diet of fresh maize kernels, maintained under air condition chamber at a temperature of 26 ± 1°C, with a photoperiod of 14 h light to 10 h dark and a relative humidity maintained of 60 ± 10%. To prevent cannibalism, each larva was isolated in an individual paper cup, with its maize supply refreshed every 48 h.

Upon reaching the pupal stage, the pupae were transferred to cylindrical cages measuring 30 cm in height and 10 cm in diameter to facilitate adult emergence. Newly metamorphosed adult males and females were provided with 10% (w/v) honey solution as a food source and allowed to mate freely in the cage environment. For oviposition, an 80-mesh nylon net was attached to the inside of the cage. Eggs laid on these nets were carefully collected and placed on a bed of fresh corn to initiate the hatching process.

### 2.2 Oviposition studies in the parental generation

Following the eclosion of *S. frugiperda*, experiments were conducted to examine the effects of sex ratio on oviposition using five different treatments. These included a normal sex ratio (2 females:2 males), two female-biased sex ratios (2 females:1 male and 3 females:1 male), and two male-biased sex ratios (1 female:2 males and 1 female:3 males). The ratios corresponded to the actual number of adult insects used in each experimental group.

For the oviposition study, moths were housed in wire mesh cages measuring 12 cm high and 11 cm in diameter, which were covered with 80-mesh nylon netting to facilitate oviposition. The nylon nets were replaced on a daily basis to ensure a clean environment for oviposition, and any moths that died were removed until the entire cohort had died. Daily observations were made to document the number of eggs laid and the time of death for each moth. This experimental protocol was repeated five times to ensure reliability and consistency of results.

### 2.3 Hatching study of the F1 generation

At the peak of oviposition in the different sex ratio treatments, a random sample of 100 eggs was collected and placed on a piece of filter paper in dishes (diameter = 9 cm). The dishes were kept humidified with sterilized water to facilitate the hatching process. After hatching, the resulting larvae were immediately transferred to fresh maize for further growth. Both the number of eggs hatched and the time taken to hatch were meticulously recorded. This procedure was repeated four times for each treatment to ensure accurate and reliable data collection (same below).

### 2.4 Study of pupation in the F1 generation

A total of one hundred newly hatched larvae, randomly selected from each established sex ratio group, were placed in a standard rectangular plastic container, 20 cm × 20 cm × 7 cm, filled with 300 g of freshly harvested maize. Third instar larvae were individually reared in paper cups that were ventilated with nylon mesh, with a fresh supply of maize provided and changed every 48 h. The pupation process was monitored for 10 days, after which the pupae were carefully collected and transferred to new paper cups for further observation. Both the number of larvae that successfully pupated and the duration of the developmental phase were meticulously documented.

### 2.5 Emergence of F1 pupae

From the pool of pupae that emerged on the same day within the different sex ratio treatments, a random sample of 50 pupae was selected and placed into individual paper cups. Each cup was lined with moistened absorbent cotton and covered with nylon gauze to maintain a moist environment conducive to emergence. The pupae were monitored daily to record the time and count the number of adult moths that emerged. Upon emergence, the adult moths were separated and followed the established rearing protocol for the F1 generation. This process was replicated consistently across all treatments, with each treatment replicated four times.

### 2.6 Egg laying in the F1 offspring

Newly emerged adult males and females of the F1 generation were randomly selected and subjected to the same conditions as the parental generation for oviposition studies. The 80-mesh nylon nets used as the oviposition substrates were refreshed daily to ensure an optimal environment for egg deposition. Deceased moths were promptly removed from the facility until the entire cohort had expired. Daily records were kept of both the number of eggs laid and the time of death for each moth. This protocol was followed for four replicates of the experiment.

### 2.7 Statistical analysis

A one-way analysis of variance was used to evaluate the effects of different sex ratio treatments on various reproductive and developmental metrics. The variables examined included the number of eggs laid per female, hatch rate, pupation rate, adult emergence rate, oviposition duration, larval stage, pupal stage, adult lifespan, and total developmental cycle duration. These analyses were performed using SPSS17 software to determine statistical significance [11]. Graphpad Prism 8.0.1 (https://www.graphpad.com/features) was used to draw the graph.

## 3 Results

### 3.1 Effects of sex ratio on the lifespan and egg production of *S. frugiperda* parental generation

**Figure 1:**
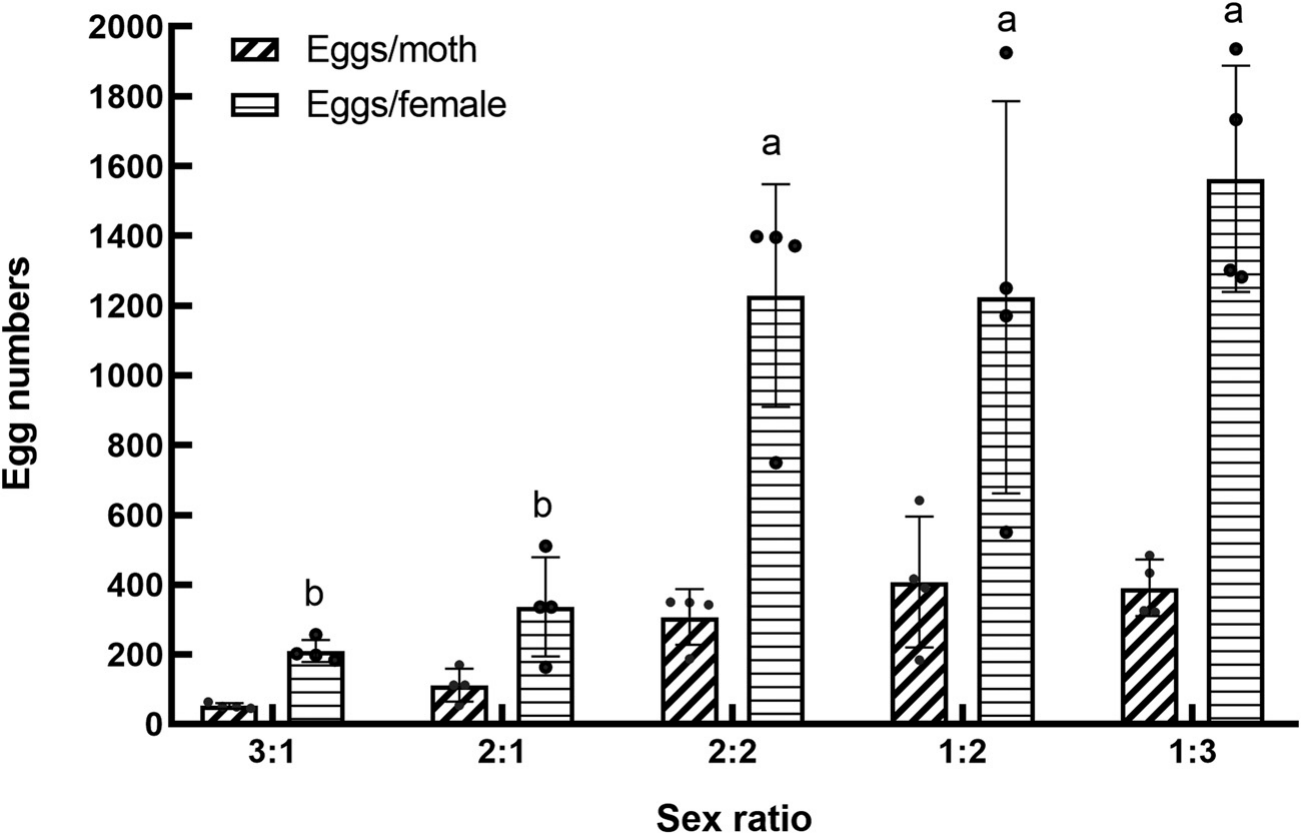
Effect of sex ratio on the egg number of parental adults of *S. frugiperda*.

In our study, the lifespan of parental adults of *S. frugiperda* did not show significant variation across various sex ratio treatments (*F*_4,15_ = 0.095, *P* > 0.05). However, the sex ratio had a pronounced effect on the fecundity of *S. frugiperda* (*F*_4,15_ = 4.819, *P* < 0.05, Figure 1). Fecundity, measured as the number of eggs per female, increased as the sex ratio became more male-biased, reaching a low at a ratio of 3:1 (159.67 eggs per female) and a high at a ratio of 1:3 (1563.5 eggs per female). The number of eggs per female at ratios of 1:3, 1:2, and 2:2 were not significantly different from each other but were significantly higher than at ratios of 3:1 and 2:1 (*P* < 0.05).

Considering that males also contribute to fertility, egg production per moth was also calculated. The analysis showed that the highest egg production per moth occurred at a sex ratio of 1:2, which was significantly higher than all other groups except for the 1:3 and 2:2 ratios (*P* < 0.05). Conversely, the lowest egg production per moth was observed at a sex ratio of 3:1, although it was not significantly different from ratios of 2:1 or 3:1.

**Figure 2:**
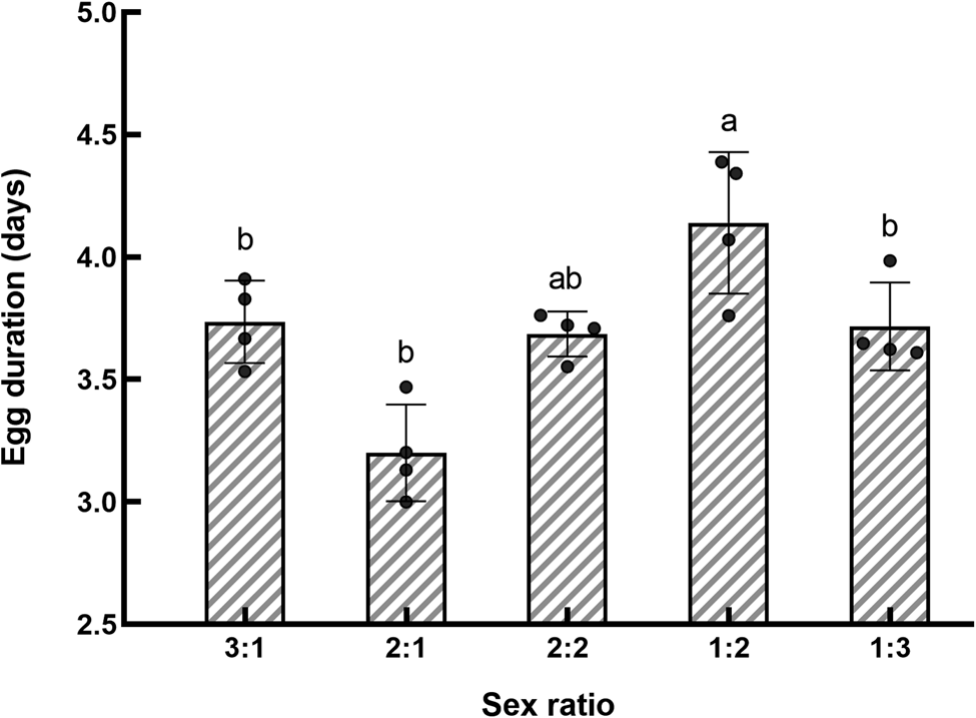
Effect of sex ratio on the egg duration in *S. frugiperda*.

**Figure 3:**
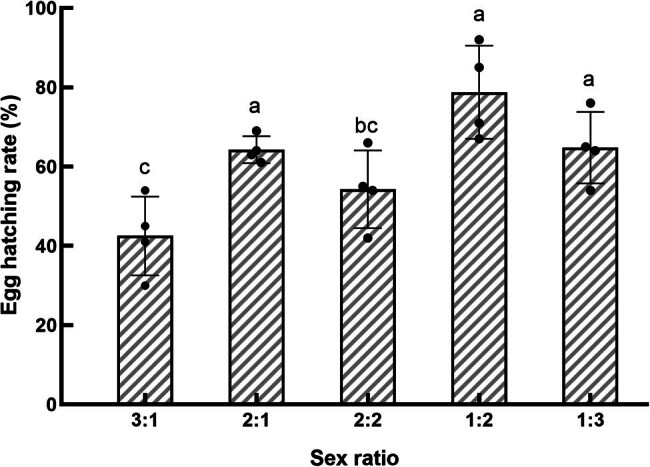
Effect of sex ratio on the hatch rate of *S. frugiperda*.

**Figure 4:**
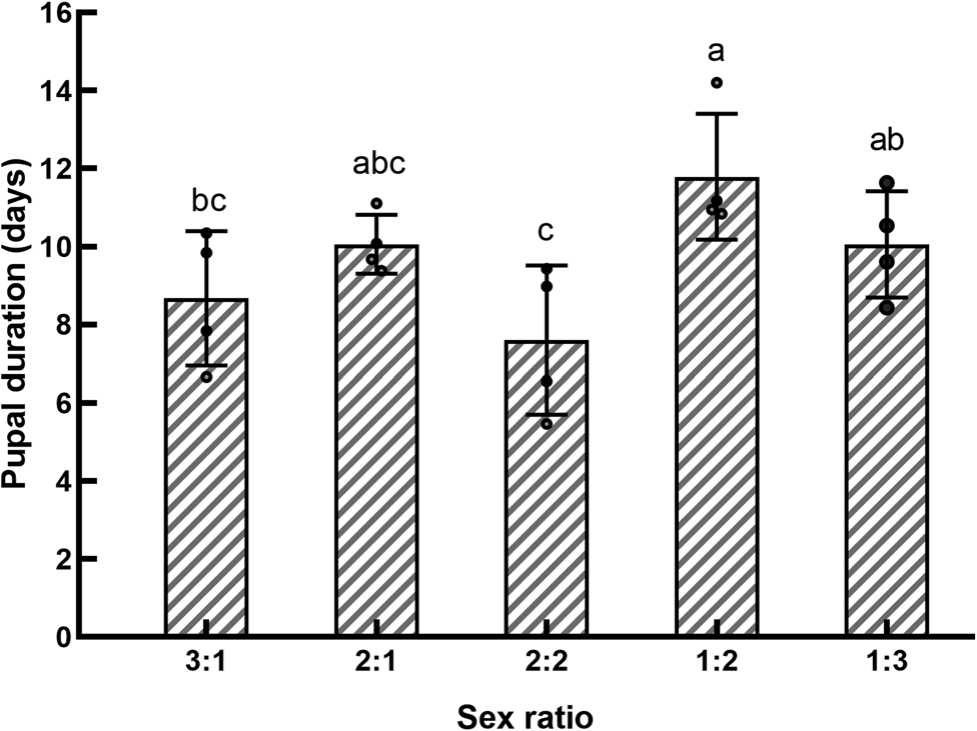
Effect of sex ratio on the pupal duration of *S. frugiperda*.

**Figure 5:**
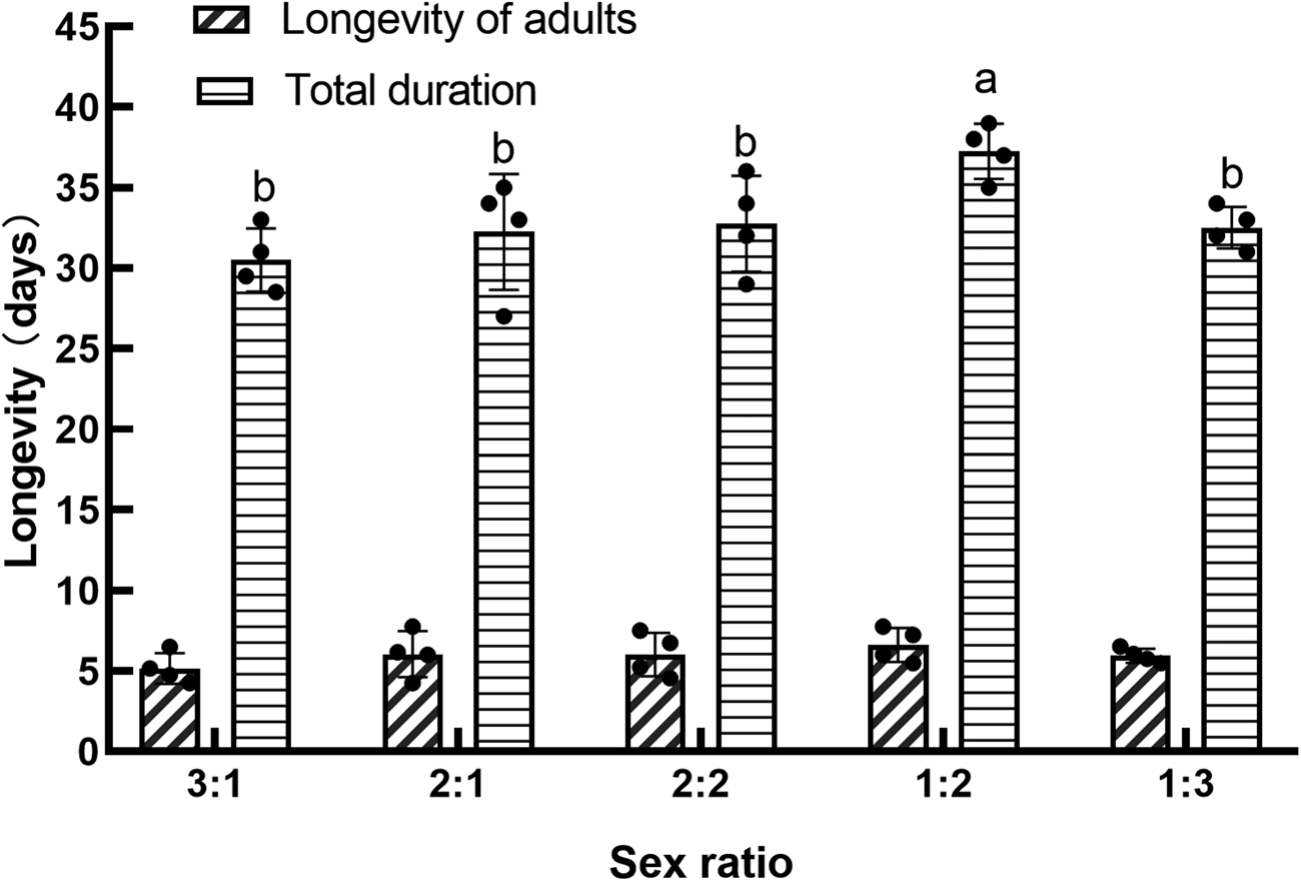
Effect of sex ratio on adult longevity and total duration of the F1 generation.

**Figure 6:**
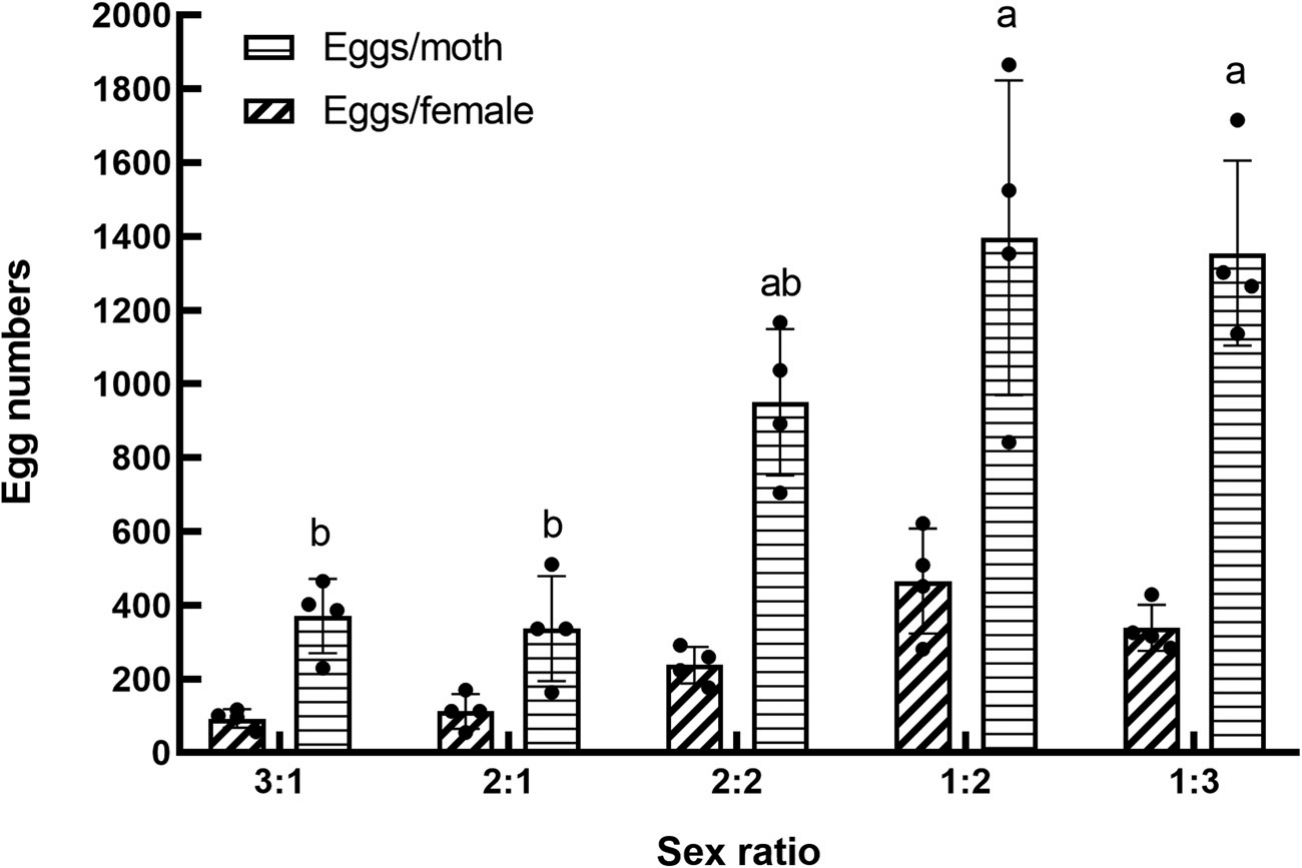
Effect of sex ratio on F1 generation egg numbers.

Data belonging to the same category and marked with different letters indicate significant differences at *P* < 0.05 lever. Same for Figures 2–6.

### 3.2 Effect of sex ratio on egg incubation time and hatchability in *S. frugiperda*

The incubation period of the egg stage of *S. frugiperda* was the longest at a sex ratio of 1:2, averaging 4.14 days. This duration was not significantly different from that observed at a 2:2 ratio (*P* > 0.05) but was significantly longer compared to the other sex ratio conditions (*P* < 0.05), as shown in Figure 2. The hatchability of the F1 generation eggs from the 1:2 sex ratio group reached the highest level at 78.8%, although this was not significantly different from the 1:3 and 2:1 sex ratio groups (*P* > 0.05), as shown in Figure 3. In contrast, the 3:1 sex ratio group had a significantly lower hatch rate of only 42.5%, which was significantly lower than the success rates observed in the other groups (*P* < 0.05).

### 3.3 Effect of sex ratio on the larval growth period and pupation success of *S. frugiperda*

The larval growth period of *S. frugiperda* was consistent across all sex ratio treatments, ranging from 13.12 to 13.82 days, with no significant variation observed (*F*_4,15_ = 1.536, *P* > 0.05). Similarly, pupation success did not show any significant differences among the different treatments (*F*_4,15_ = 0.869, *P* > 0.05).

### 3.4 Effect of sex ratio on pupal development time and adult emergence in *S. frugiperda*

The sex ratio did not significantly affect the adult emergence rate (*F*_4,15_ = 2.411, *P* > 0.05) of *S. frugiperda*. However, it had a significant effect on the pupal development time (*F*_4,15_ = 3.095, *P* < 0.05, Figure 4). The longest average pupal stage was observed in the 1:2 sex ratio, which lasted up to 11.79 days, while the shortest development time was recorded in the 2:2 sex ratio group, with an average of 8.12 days.

### 3.5 Effect of sex ratio on the reproductive output of parental *S. frugiperda* adults

The reproductive success of parental *S. frugiperda*, as measured by the number of eggs per moth and the subsequent number of moths in the F1 generation, was optimal at a sex ratio of 1:1, with the highest values for both parameters (Table 1). In contrast, the same metrics were at their lowest when the sex ratio was skewed at 3:1. These results underscore the importance of a balanced sex ratio for effective reproduction of *S. frugiperda*, suggesting that an evenly distributed male-to-female ratio is more advantageous than a population skewed towards one sex or the other.

**Table 1:** Effect of sex ratio on the life history traits of *S. frugiperda*

Reproductive parameters	Sex ratio treatments (♀︰♂)				
	3:1	2:1	2:2	1:2	1:3
Fecundity (eggs/moth)	119.8 ± 41.62c	224.3 ± 47.29bc	614.4 ± 79.93a	408.0 ± 93.75ab	322.1 ± 97.95bc
Hatching rate (%)	42.5 ± 5.0c	64.3 ± 1.7ab	56.8 ± 7.1bc	78.8 ± 5.9a	64.8 ± 4.5ab
Pupation rate (%)	59.8 ± 5.6	62.3 ± 4.3	71.5 ± 5.8	64.8 ± 8.5	58.3 ± 9.1
Emergence rate (%)	71.0 ± 6.6	79.5 ± 4.3	78.0 ± 3.4	84.5 ± 2.2	78.0 ± 2.9
Total moth	24.61 ± 9.02b	71.36 ± 16.16ab	188.04 ± 26.72a	163.58 ± 28.36ab	102.76 ± 42.63ab

Data are presented as the mean ± SE.

### 3.6 Effect of sex ratio on adult longevity and reproductive output in the F1 generation of *S. frugiperda*

Adult longevity was not significantly affected by sex ratio (*F*_4,15_ = 1.215, *P* > 0.05). However, the sex ratio had a pronounced effect on the total developmental time of the F1 generation of *S. frugiperda* (*F*_4,15_ = 3.206, *P* < 0.05), as shown in Figure 5. The treatment with a sex ratio of 1:2 resulted in the longest developmental period with an average of 37.34 days, which was significantly longer than the periods observed in the other treatment groups (*P* < 0.05).

Regarding the fecundity of the F1 generation, the sex ratio did not significantly affect the number of eggs produced per moth (*P* > 0.05). Conversely, the fecundity per female showed a significant variation between treatments (*F*_4,15_ = 3.101, *P* < 0.05, Figure 6). The highest fecundity per female was recorded in the group with a sex ratio of 1:2. This level of fecundity was comparable to that of the treatments with a sex ratio of 2:2 or 1:3 (*P* > 0.05) but was significantly higher than the fecundity observed in the other treatments (*P* < 0.05).

## 4 Discussion

FAW is an international pest; a global technical system for monitoring, early warning, and control of FAW is needed [12]. For the emergency prevention and control of *S. frugiperda*, synthetic pesticides such as pyrethroids and indoxacarb are commonly used in the field, but they also cause pest resistance after a few years and contamination of the environment and agricultural products [13,14,15]. Therefore, environmentally friendly prevention and control technologies are needed to minimize the number of FAWs in the source areas. Application of silicon by foliar spray or soil application can reduce the fecundity and pupation rate of FAW while increasing the mortality of newly emerging larvae [16]. Botanical pesticides such as matrine and azadirachtin have been most widely used in the field, while many plant extracts have been screened with high activity against FAW [17,18]. The essential oil of *Lippia sidoides* and its main compound thymol can kill the third instar larvae of FAW [19]. In addition, spore suspensions of *Metarhizium rileyi* or *Cordyceps cateniannulata* at 90% relative humidity can kill all FAW larvae when treated for 7 days [20], and releasing parasitic wasps of *Trichogramma* species against FAW in the field can reduce their egg hatching rate and greatly reduce their damage to plants [21]. Genetically modified maize, such as BT maize and food attractants on the surface of a stick object, can stick a lot of adult insects overnight and can solve the regional migration damage of FAW [22,23].

The sex ratio is an important demographic factor that can significantly affect insect reproduction and population growth dynamics, as demonstrated in studies such as Huang et al. [24]. Our research on *S. frugiperda* shows that the sex ratio affects key reproductive traits, including egg production and developmental timing. We found that a male-biased sex ratio was associated with higher fecundity and hatch rates in *S. frugiperda,* which is consistent with the idea that increased mating opportunities can lead to greater sperm and nutrient acquisition for females. This pattern of increased reproductive success with a higher number of males has been observed in other polyandrous insects, where multiple matings can increase female fitness [25]. Similar results have been found in *Assara inouei* [26], *Spodoptera exigua* [7], and *Cantheconidea furcellata* [8]. However, the effect of sex ratio on female longevity is less clear and may vary between species. In *S. frugiperda*, daily mating does not appear to reduce the lifespan of either females or males, suggesting that the cost of mating may be limited in this species. These studies provide a basis for exploring similar strategies in *S. frugiperda*, possibly using sex pheromones or other attractants to manipulate male densities and reduce offspring populations.

Sex pheromone trapping and killing technology has the advantages of low pollution, high specificity, and environmental and food safety and has become one of the important means of pest prediction and control [27]. Field studies on other Lepidoptera species, such as the gypsy moth (*Lymantria dispar*) and the codling moth (*Cydia pomonella*), have shown that pheromone disorientation and trapping can be an effective method of suppressing pest numbers [28,29]. The sex pheromones of the FAW are composed of *cis*-9-tetradecanoate and *cis*-7-dodecanoate, with the strongest attraction activity at a mass ratio of 96.6:3.4 [30]. White plastic jug traps baited with pheromones can capture 42.5% of male FAWs [31]. Releasing infertile males or using genetic modification techniques to tilt the sex ratio toward females can also reduce the overall fertility of pest populations [32].

By manipulating the sex ratio, particularly through male annihilation, as shown in the study, we can influence the reproductive success and population growth of pests. This approach can be incorporated into integrated pest management programs [33]. In addition, predicting population trends based on sex ratio effects can improve our forecasting capabilities, allowing for more proactive and timely interventions. This is particularly important for migratory pests such as FAW, where rapid population growth can lead to widespread crop damage and economic loss. Laboratory conditions differ from field conditions, and population size and density were not analyzed in the experiment. Our laboratory results need to be contextualized with field conditions, and further research is needed to assess the long-term effects of sex ratio manipulations on *S. frugiperda* populations in agricultural settings.

## 5 Conclusion

In conclusion, our study confirms that the sex ratio is a modifiable factor that can influence the reproductive success and population dynamics of *S. frugiperda.* A male-biased population appears to be more advantageous for suggesting that strategies aimed at reducing the male population, such as male annihilation, could be effective for field control. Research on the sex ratio effects of FAW contributes to the basic understanding of the biology of this pest and offers a promising avenue for the management of not only FAW, but also other insect pests.
